# Improved predictability of pancreatic ductal adenocarcinoma diagnosis using a blood immune cell biomarker panel developed from bulk mRNA sequencing and single-cell RNA-sequencing

**DOI:** 10.1007/s00262-023-03458-8

**Published:** 2023-05-10

**Authors:** Sung Ill Jang, Hyung Keun Lee, Eun-Ju Chang, Somi Kim, So Young Kim, In Young Hong, Jong Kyoung Kim, Hye Sun Lee, Juyeon Yang, Jae Hee Cho, Dong Ki Lee

**Affiliations:** 1 Department of Internal Medicine, Gangnam Severance Hospital, Yonsei University College of Medicine, 712 Eonjuro, Gangnam-gu, Seoul, 135-720 Korea; 2 Department of Ophthalmology, Institute of Vision Research, Yonsei University College of Medicine, Seoul, Korea; 3 Department of Biomedical Sciences, Asan Medical Center, University of Ulsan College of Medicine, Seoul, Korea; 4 Department of Life Sciences, Pohang University of Science and Technology (POSTECH), Pohang, Korea; 5 Biostatistics Collaboration Unit, Yonsei University College of Medicine, Seoul, Korea; 6 AccurasysBio Co., Ltd., Seoul, Korea; 7 Institute for Convergence Research and Education in Advanced Technology, Yonsei University, Seoul, Korea

**Keywords:** Pancreatic ductal adenocarcinoma, Interleukin-7 receptor, Phospholipase D4, Inhibitor of DNA binding 3, Cancer biomarkers

## Abstract

**Background:**

Pancreatic ductal adenocarcinoma (PDAC) remains a devastating cancer due to its poor survival rate, early detection, and resectability. This study aimed to determine the peripheral blood mononuclear cell (PBMC) immune biomarkers in patients with PDAC and investigate the PDAC-specific peripheral blood biomarker panel and validate its clinical performance.

**Methods:**

In this prospective, blinded, case–control study, a biomarker panel formula was generated using a development cohort—including healthy controls, patients at high risk of PDAC, and patients with benign pancreatic disease, PDAC, or other gastrointestinal malignancies—and its diagnostic performance was verified using a validation cohort, including patients with ≥ 1 lesion suspected as PDAC on computed tomography (CT).

**Results:**

RNA-sequencing of PBMCs from patients with PDAC identified three novel immune cell markers, IL-7R, PLD4, and ID3, as specific markers for PDAC. Regarding the diagnostic performance of the regression formula for the three biomarker panels, the sensitivity, specificity, positive predictive value, negative predictive value, and accuracy were 84.0%, 78.8%, 47.2%, 95.6%, and 79.8%, respectively. Based on the formula scores for the biomarker panel, the false-negative rate (FNR) of the biomarkers was 8% (95% confidence interval [CI] 3.0–13.0), which was significantly lower than that based on CT in the validation cohort (29.2%, 95% CI 20.8–37.6).

**Conclusions:**

The regression formula constructed using three PBMC biomarkers is an inexpensive, rapid, and convenient method that shows clinically useful performance for the diagnosis of PDAC. It aids diagnoses and differential diagnoses of PDAC from pancreatic disease by lowering the FNR compared to CT.

*Clinical trial registration* Clinical Research Information Service, KCT0004614 (08 January 2020).

**Supplementary Information:**

The online version contains supplementary material available at 10.1007/s00262-023-03458-8.

## Introduction

Pancreatic ductal adenocarcinoma (PDAC) is an aggressive and deadly disease with a mortality rate that closely parallels its incidence. Most studies report a mean 5-year-survival rate of < 10% [1–3] due to the fact that PDAC is resistant to therapies, and patients are diagnosed when cancer cells have already metastasized; most commonly, to the liver, lung and/or peritoneum. However, the diagnosis of PDAC lacks sensitive or specific biomarkers for early diagnosis [4, 5]. Currently used circulating biomarkers, such as CA19-9, lack sufficient sensitivity and specificity for diagnostic purposes. Therefore, establishing a non-invasive early detection method that can be used for cancer screening is important to improve the survival of patients with PDAC. However, despite numerous studies, there are still no PDAC-specific biomarkers with widespread clinical use [2, 5, 6].

Liquid biopsy holds great promise as a method for non-invasive cancer detection, particularly through the analysis of cell-free DNA, cell-free RNA fragments, extracellular vesicles (particularly exosomes), or circulating tumor cells [7, 8]. In cancer patients, these molecules and vesicles are released into the circulation by apoptosis, necrosis, and active secretion. However, the sensitive detection of usually very limited amounts of tumor-specific molecules in the blood of patients with early-stage cancers remains a challenge.

Here, we used complementary approaches, bulk RNA sequencing (RNA-seq), and single cell RNA -sequencing (scRNA-seq) to investigate the transcriptional landscape of peripheral blood mononuclear cells (PBMCs) and to determine the specific immune cell markers that may help in the differential diagnosis of PDAC from other benign pancreatic and gastrointestinal diseases. Although the peripheral blood immune landscapes of individual patients were quite heterogeneous, we selected some common PDAC-specific markers (e.g., IL-7R, PLD4, and ID3) from the blood samples of patients with PDAC that were identified to greatly contrast those of healthy controls and patients with benign pancreatic diseases, including chronic pancreatitis and cystic disease. Clinical performance was found to have remarkable value for the diagnosis and differential diagnosis of PDAC from other pancreatic diseases through examining these two cohorts. In addition, this study showcases differences in the immune landscape between PDAC and benign pancreatic diseases, which may provide a wealth of hypothesis-generating data to benefit pancreatic disease researchers.

## Materials and methods

### Study design and registration of clinical research

**Fig. 1:**
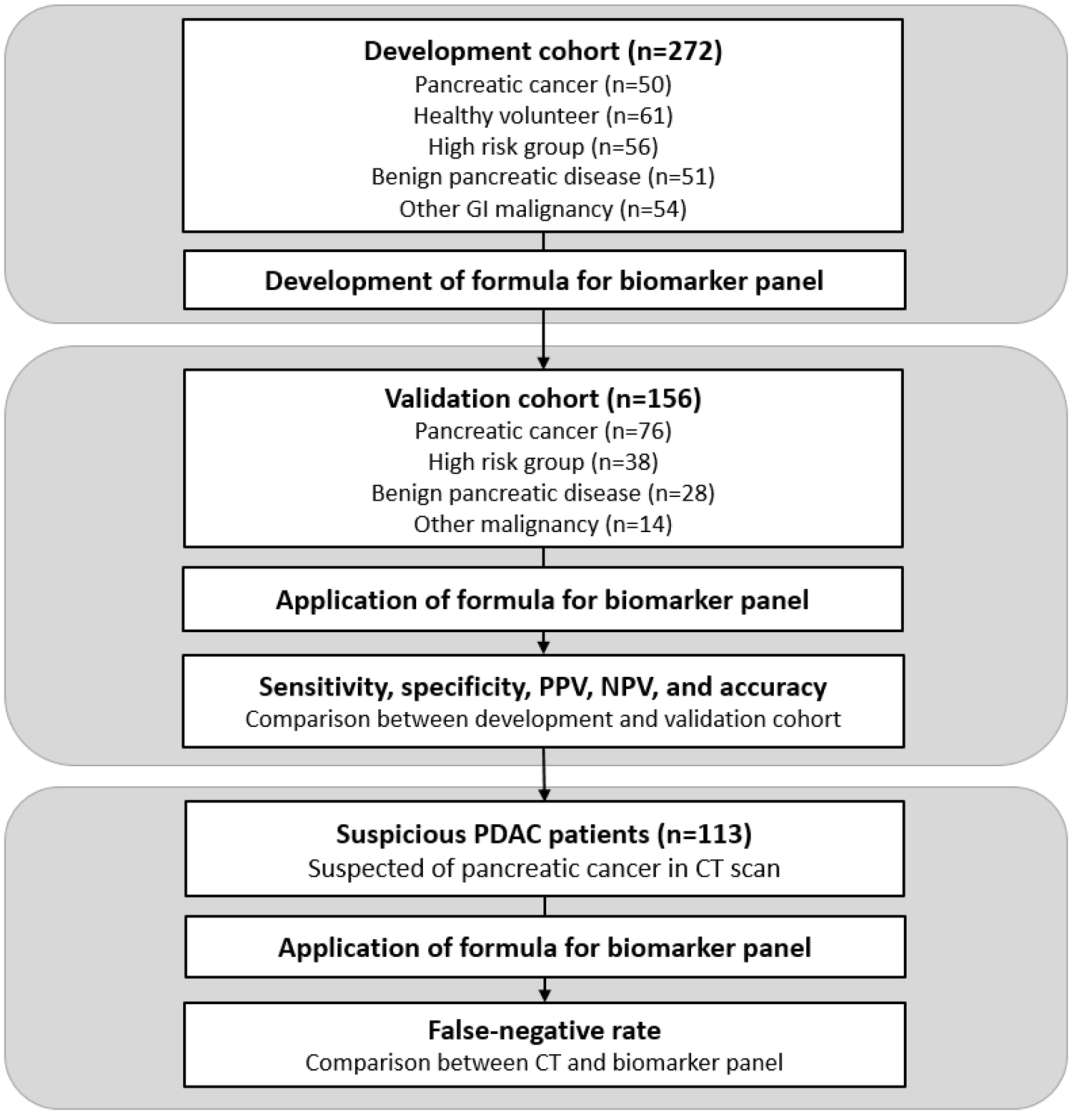
Schematic flowchart of the study design and patient flowchart. GI, gastrointestinal; CT, computed tomography; PPV, positive predictive value; NPV, negative predictive value; PDAC, pancreatic ductal adenocarcinoma

This prospective case–control study was performed using two cohorts (Fig. 1). A biomarker panel formula was created using a development cohort, and its diagnostic performance was verified using a validation cohort. The study procedures were approved by the Institutional Review Board of Gangnam Severance Hospital, Yonsei University College of Medicine, Seoul, Korea (no. 3-2018-0293 and 3-2020-0238) and registered at the Clinical Research Information Service (https://cris.nih.go.kr/cris/en/; KCT0004614, accessed on January 8, 2020). The study was conducted according to the tenets of the Declaration of Helsinki (2008, amended version), and written informed consent was obtained preoperatively from each patient and control participant.

### Patients selection and inclusion criteria of the study

The development cohort included healthy controls and patients with PDAC, high risk of PDAC, benign pancreatic disease, and other gastrointestinal (GI) malignancies. The high-risk factors for PDAC included chronic pancreatitis, pancreatic cyst, pancreatic duct dilatation, DM onset over 50 years, and elevated CA19-9 level. PDAC and other GI cancers were diagnosed by cytological examination of endoscopic ultrasound (EUS)-guided fine-needle aspiration samples or surgical specimens. Other pancreatic diseases were diagnosed by evaluating clinical symptoms and imaging studies (computed tomography [CT], EUS, and magnetic resonance imaging). Patients with evidence of serious illnesses, immunosuppression, autoimmune or infectious diseases, or those taking immunosuppressive drugs were excluded.

The inclusion criteria for the validation cohort were patients who had been diagnosed with PDAC on CT (PDAC-positive group) and those who had at least one lesion suspected as PDAC based on the results of pancreatic CT (pancreatic duct dilatation, focal alteration of parenchymal attenuation, parenchymal atrophy, pancreatic duct interruption, bile duct dilatation, double-duct sign, cystic lesion with high malignant stigma, contour abnormality of pancreatic parenchyma, and peripancreatic lymphadenopathy) in men and women aged > 19 years (PDAC-suspicious group) [9–14]. The exclusion criteria were as follows: patients for whom a blood sample could not be collected and patients with a history of immunosuppressive drug use. In addition, inappropriate blood samples were excluded for the following reasons: samples suspected of microbial contamination, improperly stored samples or storage methods that could not be confirmed, and damaged or unlabeled sample containers.

### Sample processing and DNA isolation from PBMC

Samples from all cases and controls were processed using the method described previously [15]. In brief, peripheral blood samples were collected in EDTA vacutainer tubes and processed within 3 h of collection. PBMCs were isolated from whole blood by Ficoll Paque Plus density-gradient centrifugation, according to the manufacturer’s instructions. Total RNA was extracted from PBMCs using QIAzol (QIAZEN) and reverse-transcribed to cDNA using PrimeScript RT Master Mix (TaKaRa). For quality control of the yielded RNA, the purity and integrity of RNA were evaluated by OD 260/280 ratio and analyzed using the Agilent 2100 Bioanalyzer (Agilent Technologies, Palo Alto, CA, USA).

### mRNA collection and quantitative RT-PCR

Quantitative real-time PCR was performed using a PCR detection system (StepOnePlus Real-Time PCR; Applied Biosystems) and commercial detection kit (Taqman™ Gene expression Master Mix; Applied Biosystems) according to the manufacturer's instructions. The amplification program included an initial denaturation step at 95 °C for 10 min, followed by 40 cycles of denaturation at 95 °C for 15 s and annealing and extension at 60 °C for 60 s. Primer sequences used in this study are listed in Supplementary Table 1. Quantitative PCR (qPCR) results were analyzed using the comparative Ct method and normalized to glyceraldehyde 3-phosphate dehydrogenase levels.

### mRNA sequencing (mRNA-seq) and data analysis

The Affymetrix whole-transcript expression array process was performed according to the manufacturer’s instructions (GeneChip Whole Transcript PLUS reagent Kit). cDNA was synthesized using the GeneChip Whole Transcript (WT) Amplification Kit, according to the manufacturer's instructions. Sense cDNA was then fragmented and biotin-labeled with terminal deoxynucleotidyl transferase using the GeneChip WT Terminal labeling kit. Approximately 5.5 μg of labeled DNA was hybridized to the Affymetrix GeneChip human 2.0 ST Array at 45 °C for 16 h. Hybridized arrays were washed and stained on GeneChip Fluidics Station 450 and scanned using the GCS3000 scanner (Affymetrix). Signal values were computed using the Affymetrix® GeneChip™ Command Console software.

### Bulk RNA-seq data analysis

Raw read counts were normalized and log_2_ fold changes (log2FC) of genes between conditions were calculated by using the DESeq function of the DESeq2 (v1.34.0) R package [16] with a design formula considering two factor variables: condition and sex. To rank and visualize the effect size of genes between conditions effectively, shrinkage of effect size was calculated per each gene by using the lfcShrink function of the same package. For deconvolution analysis, protein coding genes were used, except for mitochondrial, ribosomal, and gonosomal genes. The posterior sum over different cell types defined by the published scRNA-seq data [17] was calculated by using the run.prism function of the BayesPrism (v2.0) R package [18] with default parameters.

### scRNA-seq data analysis

Count matrices for 4 human normal PBMC scRNA-seq data were downloaded [17]. Poor-quality cells with log 10-scaled counts < 2.5 and percentage of UMIs mapped to mitochondria > 20 were discarded using the percellQCMetrics function of the scater (v1.18.6) R package [19], and a total of 18,895 cells from 4 samples were used for further analysis. Raw UMI counts were normalized in log2-scale using the logNormCounts function of scran (v1.18.7) R package [20] after removing cell-specific biases by clustering cells and calculating cell-specific size factors using the quickCluster and the computeSumFactors of the same R package. After decomposing gene-specific variance into biological and technical components using the modelGeneVar function of the same R package, highly variable genes (HVGs) were identified with FDR < 0.05. The top 20 PCs with HVGs were calculated for further cell clustering and visualization. A Shared Nearest Neighbor (SNN) graph was constructed using the FindNeighbors function of Seurat (v4.2.0) R package [21] with the default parameters. Cells were visualized on the two-dimensional UMAP plot using the RunUMAP function. After cell type annotation using expression of canonical markers based on the reference paper to compare relative gene expression among cell types, the normalized counts were scaled, and the scaled expression was averaged per each cell type.

### Immunohistochemical staining

Immunohistochemical staining (IHC) for PLD4 and ID3 was performed on 5-μm histological sections that were cut from the TMA blocks. PLD4/CD20 and ID3/CD3 immunohistochemical double staining was carried out using a BenchMark Ultra IHC/ISH System (Ventana Medical Systems, Tucson, Az) according to the manufacturer’s instructions. Antibodies for IHC are listed in Supplementary Table 2.

### Statistical analysis

Using the development dataset, the study population characteristics are presented as mean ± standard deviation for continuous variables and frequencies (percentages) for categorical variables. Differences between groups were analyzed using one-way analysis of variance for continuous variables and chi-square tests for categorical variables. Post hoc analyses were conducted using the Bonferroni method. Univariate logistic regression analysis was performed to evaluate the association between pancreatic cancer and CA19-9, IL-7R, PLD4, and ID3 levels. To construct the biomarker panel, multivariate logistic regression was performed to identify independent factors, including IL-7R, PLD4, and ID3. Optimal cutoff values for CA19-9, IL-7R, PLD4, ID3, and the biomarker panel were determined by calculating Youden’s index.

In the development and validation datasets, we assessed the performance of CT findings, CA19-9, IL-7R, PLD4, ID3, and the biomarker panel. Diagnostic performance was evaluated based on sensitivity, specificity, accuracy, positive predictive values (PPVs), negative predictive values (NPVs), and false-negative rate (FNR) values. In this study, we used two definitions of FNR. For the development and validation datasets with both positive and negative CT findings, we used the classic FNR definition (FN/[TP + FN]). For the validation dataset with only negative CT, we defined the modified FNR as the percentage of FN in patients with negative CT findings (FN/total N). Additionally, receiver operating characteristic (ROC) curves were constructed, and AUCs were calculated. A generalized estimation equation was used to compare the diagnostic performance.

Statistical analyses were performed using SAS (version 9.4; SAS Institute, Cary, NC, USA) and R (version 4.0.3; http://www.R-project.org, accessed on January 2, 2020). The significance level was set at a *p*-value of < 0.05.

## Results

### Selection of PDAC-specific PBMC biomarkers from RNA-seq data

**Fig. 2:**
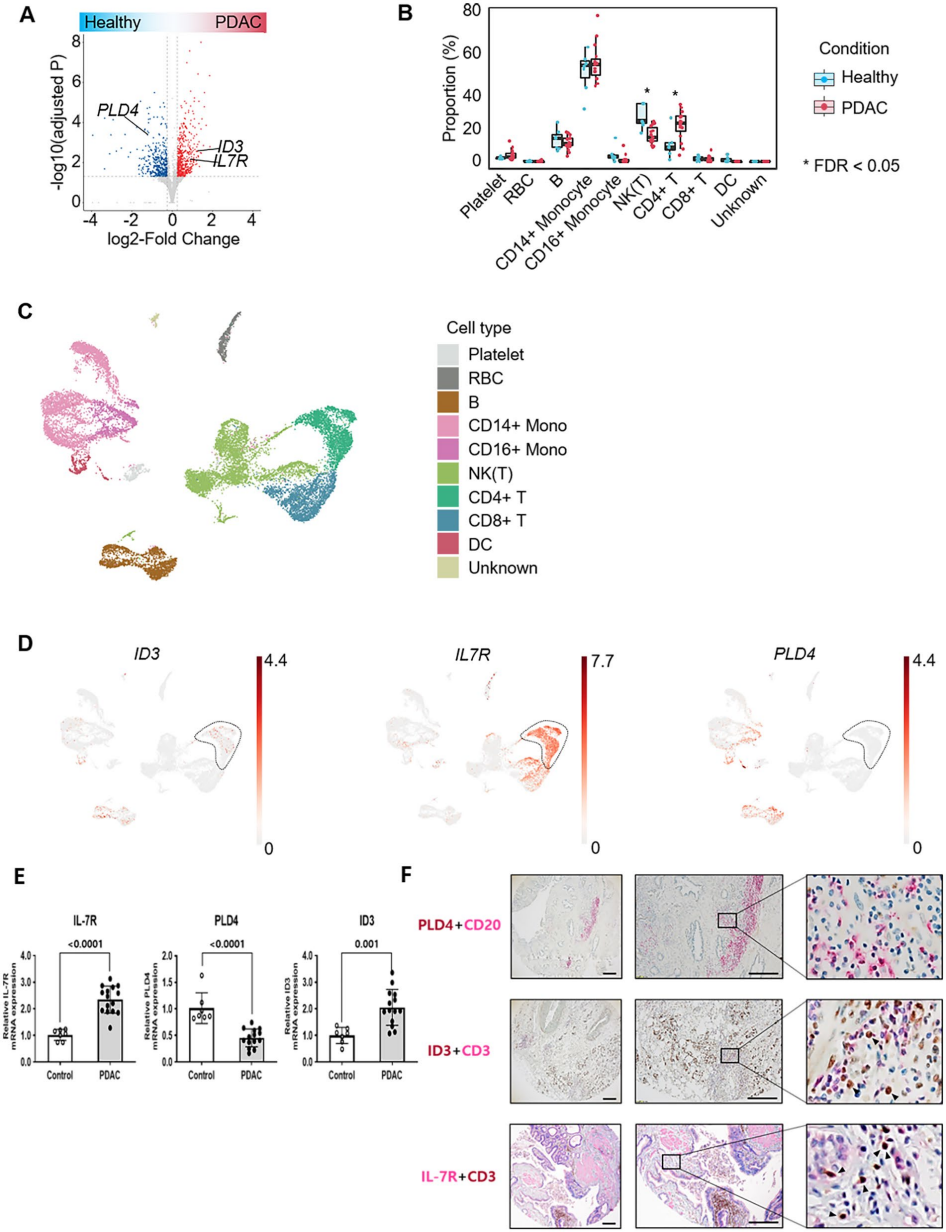
Determination of PDAC-specific PBMC marker selection. **A**. Volcano plot depicting DEGs between patients with PDAC and controls. Blue and red dots are genes satisfying adjusted *p*-value < 0.05 and log2 fold change < − 0.25 and > 0.25, respectively. **B**. Box plot showing inferred cell type composition in RNA-seq in 15 patients with PDAC and 7 controls. **C**. UMAP plot of 18,895 cells from the PBMCs of four healthy controls. **D**. UMAP plot showing expression of ID3, IL-7R, and PLD4. Black dotted lines highlight CD4 T cells. **E**. qPCR data for IL-7R, PLD4, and ID3 between controls and patients with PDAC. **F**. Immunohistochemical staining of ID3-expressing cells and IL-7R-expressing cells. PLD4-expressing immune cells were not found; however, CD3 + IL-7R + and CD3^+^ID3^+^-co-expressing cells were found in PDAC tissues. PDAC, pancreatic ductal adenocarcinoma; PBMC, peripheral blood mononuclear cells; DEGs, differentially expressed genes; IL-7R, interleukin-7 receptor; PLD4, phospholipase D 4; ID3, inhibitor of DNA binding 3

To screen candidate PDAC-specific immunological biomarkers from blood samples, we performed RNA-seq on PBMC samples from 15 patients with PDAC and 7 healthy controls. We identified 616 upregulated and 415 downregulated genes in patients with PDAC compared to controls (Fig. 2A). To correlate these DEGs with changes in cell type composition in patients with PDAC, we re-analyzed the published human PBMC scRNA-seq dataset as a single-cell reference. Based on 9 distinct cell types and 1 unknown cluster identified from the scRNA-seq data (Fig. 2C), we deconvolved each bulk RNA-seq sample into 10 cell types using BayersPrism. Compared with the control group, the analysis showed that CD4 T cells were significantly expanded in patients with PDAC (Fig. 2B and Supplementary Fig. 1), suggesting that the expansion of CD4 T cells is important in explaining the observed DEGs between healthy patients and those with PDAC. Therefore, we selected two genes that are upregulated in patients with PDAC and expressed in CD4 T cells (IL-7R and ID3) as positive markers. As a negative marker, we selected *PLD4*, which is downregulated in patients with PDAC and not expressed in CD4 T cells (Fig. 2D).

To determine whether the selected markers could more accurately discriminate PDAC from other benign pancreatic diseases and controls, we determined the mRNA expression levels of these genes using qPCR in 120 patients. qPCR assays of each marker—IL-7R, PLD4, and ID3—revealed them as novel PDAC markers (Fig. 2E). The expression of IL-7R and its functional role have been recently reported [15, 22]. The qPCR assay showed that PLD4 was downregulated and ID3 was significantly upregulated in the PBMCs of patients with PDAC. In addition, we confirmed that PLD4- and ID3-expressing immune cells infiltrated PDAC tissues (Fig. 2F).

### Demographic and laboratory data of the developing cohort population

**Fig. 3:**
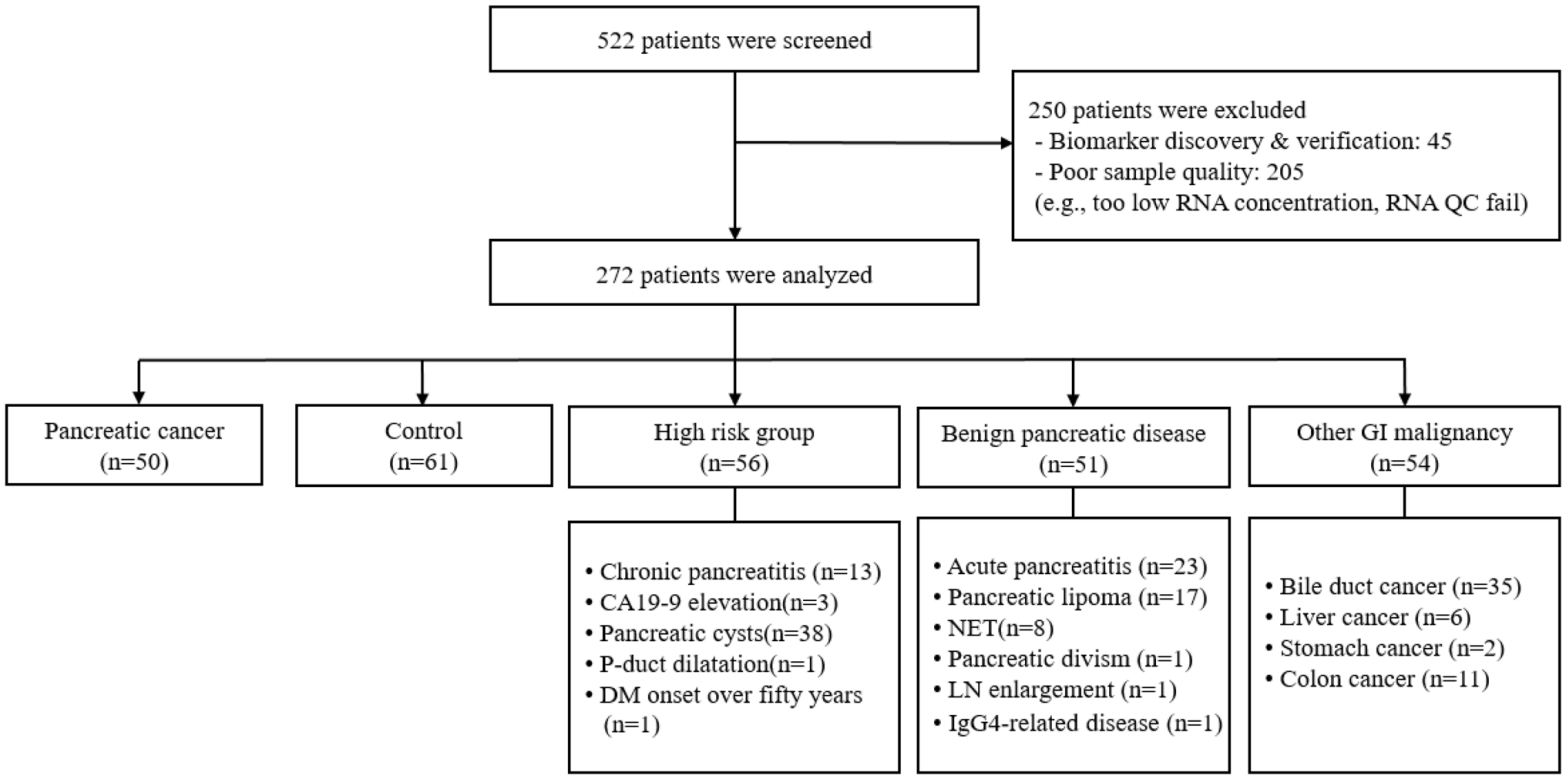
Population data for the development cohort. QC, quality control; P-duct, pancreatic duct; DM, diabetes mellitus; NET, neuroendocrine tumor; LN, lymph node; GI, gastrointestinal

To determine the clinical performance of these three markers, we constructed a clinical cohort. Of the 552 screened patients, 250 were excluded (e.g., due to incomplete dataset), and 272 patients were enrolled in the development cohort and their data were analyzed (Fig. 3). These patients were classified into five groups: PDAC (*n* = 50), healthy controls (*n* = 61), high-risk group (*n* = 56), benign pancreatic disease (*n* = 51), and other GI malignancies (*n* = 54). Demographic characteristics were not significantly different between the PDAC, healthy controls, high-risk, benign pancreatic disease, and other GI malignancy groups, except that the PDAC group and other GI malignancy groups were older than the other groups (Supplementary Table 3). Bilirubin, aspartate transaminase, alanine transferase, and CA19-9 levels were higher in the PDAC group than in the other groups owing to biliary obstruction by PDAC. CA19-9 levels were also higher in the PDAC group than in the other groups.

Among the selected biomarkers, IL-7R mRNA levels were significantly higher in the PDAC group than in the other groups. PLD4 mRNA levels were significantly downregulated and ID3 mRNA levels were significantly higher in the PDAC group than in the other groups.

### Formula and diagnostic performance of the biomarker panel

The expression of CA19-9 in serum and mRNA level of three markers (IL-7R, PLD4, and ID3) in PBMC was significantly different between PDAC and non-PDAC (Supplementary Table 4). Furthermore, the IL-7R, PLD4, and ID3 markers were statistically significant both in the univariate and multivariate models for differentiating pancreatic cancer from non-pancreatic cancer. Therefore, combinations of these three biomarkers were used to establish the formula for the biomarker panel.

$$\mathrm{A}=-0.789445-0.229438\times \left(\mathrm{PLD}4\right)+0.001251\times \left(\mathrm{IL}7\mathrm{R}\right)+0.134571\times \left(\mathrm{ID}3\right)$$

$$\mathrm{Pr}\left(Y=\mathrm{pancreatic cancer}\right)=\frac{1}{1+\mathrm{exp}(-A)}$$

**Fig. 4:**
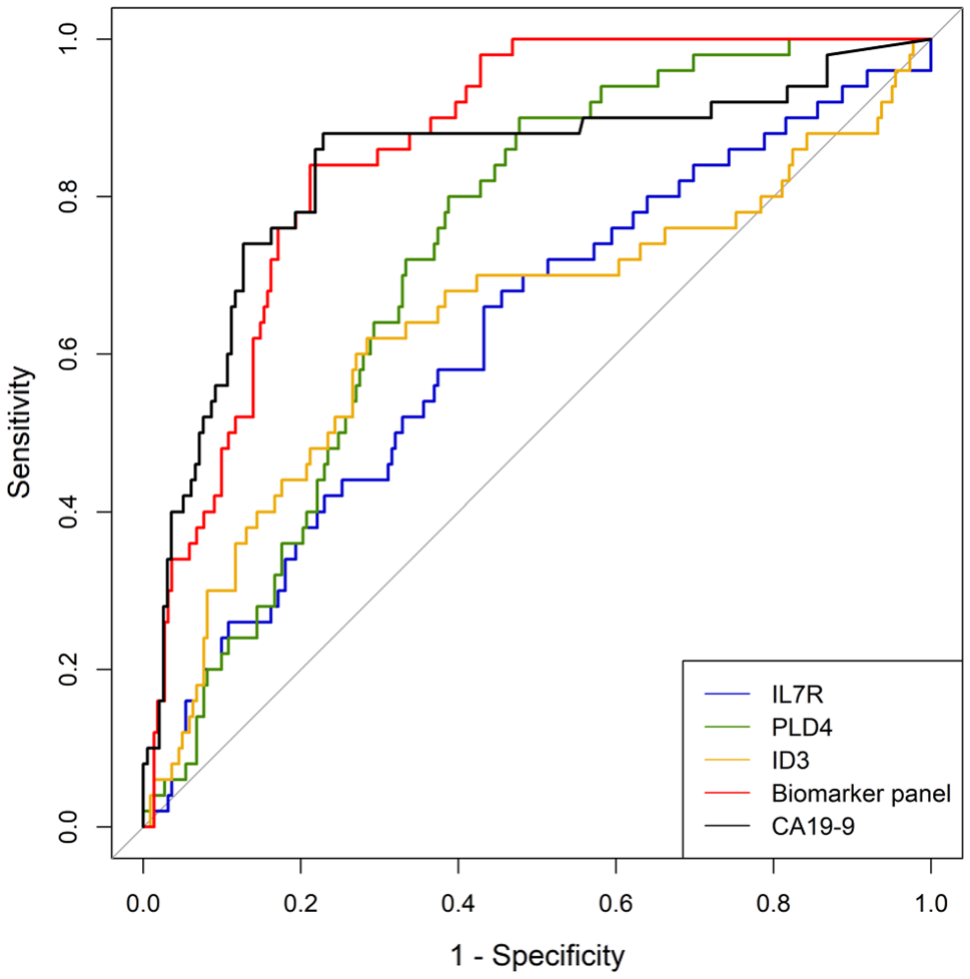
ROC curves for CA19-9, PLD4, IL-7R, ID3, and the biomarker panel for PDAC assessment. The AUC for the biomarker panel was significantly higher than that for IL-7R, PDL4, and ID3 individually. The AUC for CA19-9 did not differ from that of the biomarker panel. ROC, receiver operating characteristics; PDAC, pancreatic ductal adenocarcinoma; AUC, area under the curve; CA19-9, carbohydrate antigen 19–9; IL-7R, interleukin-7 receptor; PLD4, phospholipase D 4; ID3, inhibitor of DNA binding 3

The AUC for the biomarker panel was significantly higher than that for IL-7R, PLD4, and ID3 (Fig. 4). However, the AUC for CA19-9 was not different from that of the biomarker panel. The diagnostic performance of the biomarker panel was superior to that of each marker alone in the development cohort (Table 1). The sensitivity, specificity, PPV, NPV, and accuracy of the biomarker panel were 84.0%, 78.8%, 47.2%, 95.6%, and 79.8%, respectively. Notably, these values were significantly higher than those for IL-7R (66%, 56.8%, 25.6%, 88.1%, and 58.5%, respectively), PLD4 (90%, 52.3%, 29.8%, 95.9%, and 59.2%, respectively), and ID3 (62%, 71.6%, 33%, 89.3%, and 69.9%, respectively) alone (*p* < 0.001).

**Table 1:** Diagnostic performance of multi-panel biomarkers and CA19-9 for pancreatic adenocarcinoma in development cohort

Marker	Sensitivity (95% CI)	Specificity (95% CI)	Accuracy (95% CI)	PPV (95% CI)	NPV (95% CI)	Classic FNR^†^ (95% CI)	AUC (95% CI)	*p*-value^‡^
CT	48.0 (34.2–61.8)	100.0 (100.0–100.0)	90.4 (86.9–93.9)	100.0 (100.0–100.0)	89.5 (85.7–93.3)	52.0 (38.2–65.8)	74.0 (67.0–81.0)	< .0001
CA19-9	76.0 (64.2–87.8)	82.2 (76.9–87.5)	81.0 (76.1–85.8)	51.4 (40.0–62.7)	93.3 (89.6–96.9)	24.0 (12.2–35.8)	79.1 (72.6–85.6)	0.2795
IL-7R	66.0 (52.9–79.1)	56.8 (50.2–63.3)	58.5 (52.6–64.3)	25.6 (18.1–33.1)	88.1 (82.8–93.4)	34.0 (20.9–47.1)	61.4 (54.0–68.8)	0.0217
PLD4	90.0 (81.7–98.3)	52.3 (45.7–58.8)	59.2 (53.4–65.0)	29.8 (22.5–37.1)	95.9 (92.3–99.4)	10.0 (1.7–18.3)	71.1 (65.8–76.5)	0.3618
ID3	62.0 (48.5–75.5)	71.6 (65.7–77.6)	69.9 (64.4–75.3)	33.0 (23.5–42.5)	89.3 (84.8–93.9)	38.0 (24.5–51.5)	66.8 (59.4–74.2)	0.0107
Biomarker panel	84.0 (73.8–94.2)	78.8 (73.5–84.2)	79.8 (75.0–84.6)	47.2 (36.8–57.6)	95.6 (92.7–98.6)	16.0 (5.8–26.2)	81.4 (75.6–87.2)	Reference

Values are % (95% confidence interval)

Cut-off points: CA19-9 > 37.0, IL-7R > 1025.40733, PLD4 < 18.8693, ID3 > 8.7021, Multi-panel > 0.22016

CT, computed tomography; PPV, positive predictive value; NPV, negative predictive value; FNR, false negative rate; AUC, area under curve; CA19-9, carbohydrate antigen 19–9; IL-7R, interleukin-7 receptor, PLD4, phospholipase D4; ID3, inhibitor of DNA binding 3

^†^, Classic FNR calculated False Negative/(True Positive + False Negative)

^‡^, *p*−value compared with multi−panel biomarker for FNR

### Patient characteristics and diagnostic performance in the validation cohort

**Fig. 5:**
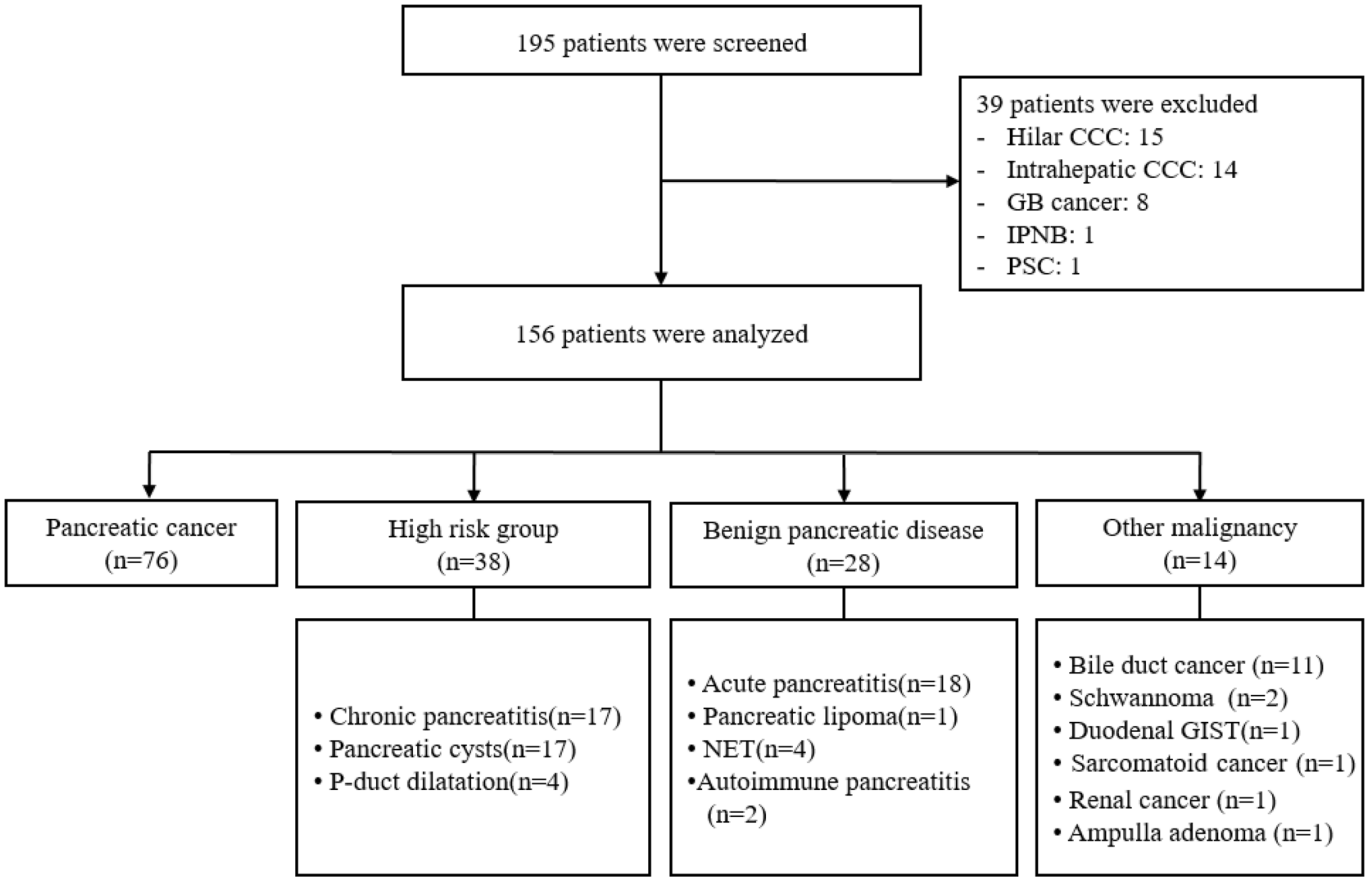
Population data for the validation cohort. CCC, cholangiocarcinoma; GB, gallbladder; IPNB, intraductal papillary neoplasm, biliary; PSC, primary sclerosing cholangitis; NET, neuroendocrine tumor; GIST, gastrointestinal stromal tumor

Of the 195 screened patients, 39 were excluded, and 156 patients were enrolled in the validation cohort and their data were analyzed (Fig. 5). These patients included those with PDAC (*n* = 76), high-risk of PDAC (*n* = 38), benign pancreatic disease (*n* = 28), and other malignancies (*n* = 14). Patients were classified into PDAC-positive (*n* = 43) and PDAC-suspicious (*n* = 113) groups according to the CT results (Supplementary Table 5). There were no differences in patient characteristics between the two groups, except that the biomarker panel levels of the PDAC-positive group were significantly higher than those of the PDAC-suspicious group (*p* = 0.001). The proportion of patients diagnosed with PDAC was 100% (43/43) in the PDAC-positive group and 29.2% (33/113) in the PDAC-suspicious group.

The diagnostic performance of the biomarker panel in the validation cohort did not differ from that in the development cohort (Table 2). The sensitivity, specificity, PPV, NPV, and accuracy for the biomarker panel were 80.3%, 78.8%, 78.2%, 80.8%, and 79.5%, respectively. However, the diagnostic performance in terms of sensitivity of CA19-9 in the validation cohort was lower than that in the development cohort (56.6 vs. 76.0).

**Table 2:** Diagnostic performance of multi-panel biomarkers and CA19-9 for pancreatic adenocarcinoma in validation cohort

Marker	Sensitivity (95% CI)	Specificity (95% CI)	Accuracy (95% CI)	PPV (95% CI)	NPV (95% CI)	Classic FNR^†^ (95% CI)	AUC (95% CI)	*p*-value^‡^
CT	56.6 (45.4–67.7)	100.0 (100.0–100.0)	78.8 (72.4–85.3)	100.0 (100.0–100.0)	70.8 (62.4–79.2)	43.4 (32.3–54.6)	78.3 (72.7–83.9)	0.0004
CA19-9	68.4 (58.0–78.9)	77.5 (68.3–86.7)	73.1 (66.1–80.0)	74.3 (64.0–84.5)	72.1 (62.6–81.6)	31.6 (21.1–42.0)	73.0 (66.0–80.0)	0.0658
IL-7R	52.6 (41.4–63.9)	52.5 (41.6–63.4)	52.6 (44.7–60.4)	51.3 (40.2–62.4)	53.8 (42.8–64.9)	47.4 (36.1–58.6)	52.6 (44.7–60.5)	0.0001
PLD4	75.0 (65.3–84.7)	34.2 (23.7–44.6)	54.2 (46.3–62.0)	52.3 (42.9–61.7)	58.7 (44.5–72.9)	25.0 (15.3–34.7)	54.6 (47.4–61.8)	0.3141
ID3	50.0 (38.8–61.2)	47.5 (36.6–58.4)	48.7 (40.9–56.6)	47.5 (36.6–58.4)	50.0 (38.8–61.2)	50.0 (38.8–61.2)	51.3 (43.4–59.1)	< .0001
Biomarker panel	80.3 (71.3–89.2)	78.8 (69.8–87.7)	79.5 (73.2–85.8)	78.2 (69.0–87.4)	80.8 (72.0–89.5)	19.7 (10.8–28.7)	79.5 (73.1–85.9)	Reference

Values are % (95% confidence interval)

Cut-off points: CA19-9 > 37.0, IL-7R > 1025.40733, PLD4 < 18.8693, ID3 > 8.7021, Multi-panel > 0.22016

PPV, positive predictive value; NPV, negative predictive value; FNR, false negative rate; AUC, area under curve; CA19-9, carbohydrate antigen 19–9; IL-7R, interleukin-7 receptor, PLD4, phospholipase D4; ID3, inhibitor of DNA binding 3

^†^,Classic FNR calculated False Negative/(True Positive + False Negative)

^‡^, *p*−value compared with multi−panel biomarker for FNR

### Abnormal findings on abdominal CT in PDAC-suspicious group

Abnormal findings on abdominal CT in patients in the validation cohort are summarized in Supplementary Table 6. The most common abnormal CT finding was focal alteration of parenchymal attenuation, which was identified in 76 patients (67.3%). Other findings included pancreatic duct dilatation in 39 patients, cystic lesions with high malignant stigma in 28 patients, parenchymal atrophy in 27 patients, contour abnormality of the pancreatic parenchyma in 18 patients, peripancreatic lymphadenopathy in 18 patients, bile duct dilatation in 12 patients, double-duct sign in 12 patients, and pancreatic duct interruption in 1 patient. There were cases of multiple abnormal CT findings in one patient, and the mean number of findings was 2.04 ± 0.87.

### Application of the biomarker panel in PDAC-suspicious group

Based on the formula scores for the biomarker panel, the 113 patients with suspected PDAC on abdominal CT were divided into high-risk (positive, Pr ≥ 0.22016) and low-risk (negative, Pr < 0.22016) groups. Of the 41 high-risk cases, 24 (58.5%) were PDAC cases and 17 (41.5%) were benign cases. The 72 low-risk cases included 9 (12.5%) PDAC cases and 63 (87.5%) benign cases. The sensitivity, specificity, PPV, NPV, and accuracy for the biomarker panel were 72.7%, 78.8%, 58.5%, 87.5%, and 77%, respectively (Table 3). Among the 113 patients who were negative for PDAC based on CT, 33 patients were diagnosed with PDAC. Therefore, the FNR of CT was 29.2% (95% confidence interval [CI] 20.8–37.6). Nine patients with PDAC were diagnosed from the 113 patients who tested negative based on the biomarker panel, and the FNR of the biomarkers was 8% (95% CI 3.0–13.0), which was 72.4% lower than the FNR of CT (*p* < 0.001).

**Table 3:** Diagnostic performance of multi-panel biomarkers and CA19-9 for pancreatic adenocarcinoma in suspicious PDAC group

Marker	Sensitivity (95% CI)	Specificity (95% CI)	Accuracy (95% CI)	PPV (95% CI)	NPV (95% CI)	Modified FNR^†^ (95% CI)	AUC (95% CI)	*p*-value^‡^
CT	NA	NA	NA	NA	70.8 (62.4–79.2)	29.2 (20.8–37.6)	NA	< 0.0001
CA19-9	63.6 (47.2–80.0)	77.5 (68.3–86.7)	73.5 (65.3–81.6)	53.8 (38.2–69.5)	83.8 (75.4–92.2)	10.6 (4.9–16.3)	70.6 (61.0–80.1)	0.4039
Biomarkers panel	72.7 (57.5–87.9)	78.8 (69.8–87.7)	77.0 (69.2–84.8)	58.5 (43.5–73.6)	87.5 (79.9–95.1)	8.0 (3.0–13.0)	75.7 (66.8–84.7)	Reference

Values are % (95% confidence interval)

Cut-off points: CA19-9 > 37.0, Biomarkers panel > 0.22016

PPV, positive predictive value; NPV, negative predictive value; FNR, False negative rate; AUC, area under curve; CA19-9, carbohydrate antigen 19–9; CT, computed tomography; NA, non-available

^†^, Modified FNR calculated False Negative/Total N

^‡^, *p*−value compared with biomarkers panel for FNR

## Discussion

Molecular biomarkers for cancer diagnosis can be classified as direct or indirect. Direct biomarkers are related to or are segments of tumor tissues (e.g., tumor DNA and RNA). However, indirect biomarkers could be reminiscent of known or unknown factors implicated in the dysregulation of cell functions. PBMCs have emerged as a novel source of biomarkers in various disorders, including inflammatory diseases and cancers [23–25]. PBMCs may mimic the conditions of some tissues they are in direct contact with, such as tumor cells [24]. Recent experiments have shown that gene expression and methylation profiles in PBMCs are altered in the presence of malignancies, such as non-small-cell lung cancer, renal cell carcinoma, breast cancer, and other cancers. [24, 26, 27] In light of this idea, we investigated peripheral blood markers for cancer detection using recent sophisticated detection tools (RNA-seq and scRNA-seq).

Local paracrine effects and crosstalk between tumor cells and immune cells are important factors in tumor growth and suppression; however, immunity is coordinated across tissues. For example, many myeloid cells are frequently replenished from hematopoietic precursors in the bone marrow [28], and critical T-cell priming events typically occur in lymphoid tissues [29]. Recent clinical and preclinical studies are unravelling the range of systemic immune perturbations that occur during tumor development, as well as the crucial contribution of peripheral immune cells to an anti-tumor immune response. Therefore, we sought to identify immune biomarkers for PDAC. Although biomarkers are found in the blood, their detection remains challenging due to the high degree of fragmentation, minute quantity, and large amount of non-specific background [7, 8]. In this context, we attempted to find a more stable and intact source of biomarkers and identified three markers from PBMCs as a target source for biomarker detection [25, 30].

IL-7R is a well-known marker for some T cells, including naive and stem cell memory T cells [31–33]. Our team recently reported that the IL-7R level is elevated in blood cells from patients with PDAC, especially in the early stages of the disease. Although this study focused on the detection and validation of biomarkers, the molecular and biological mechanisms were not investigated. However, considering the hypothesis of tumor immune surveillance [34], it is reasonable that the IL-7R level in T cells is elevated in the early period of PDAC. From the scRNA-seq data, PLD4 was highly expressed in B cells and monocytes from patients with PBMC (Fig. 2B). Although not much data have been published on the role of PLD4 in cancer, PLD4 has been reported as a critical factor for tumor as it plays an important role in anti-tumor activities in colon cancer [35] and renal fibrosis [36]. To the best of our knowledge, no study has reported the role of PLD4 in PDAC development. However, we found that PLD4 levels were downregulated in PDAC and well correlated with tumor stage (data not shown). ID3 is a member of the ID family of helix-loop-helix proteins and lacks a basic DNA-binding domain that is known to inhibit transcription. ID3 is highly expressed in B and T cells [37, 38], especially Th1 type cells [38] and tissue-resident regulatory T cells [39]. Similar to previous studies, ID3 was significantly elevated in T and B plasma cells in PMBCs from patients with PDAC. Few studies have investigated the role of PLD4 and ID3 in tumor growth and inhibition, especially PDAC. Therefore, future studies investigating the precise expression pattern (e.g., expression pattern with tumor growth) and functional role of those markers (e.g., prognostic role) are required.

We believe that these three indirect tumor markers have three clinical values. First, the biomarker panel can improve the FNR of abdominal CT and CA19-9 level; the combination of CT and the biomarker panel reduced the FNR to 8.0% (Table 3). Considering most patients with suspected PDAC had undergone abdominal CT, it is impressive that this simple and 1-day blood cell examination significantly improved diagnostic value. Second, these marker levels were selectively higher in relatively early cases that did not have elevated CA19-9 levels. Using qPCR data from these markers, 21 (42.3%) CA19-9-negative cancer cases were clinically confirmed as PDAC cases. It is well-known that CA19-9 levels are elevated in late-stage disease and correlate with mass size [40–42]. Therefore, it is meaningful that the three-marker test positively identified non-diagnostic cases based on CA19-9 level and abdominal CT findings. Although the survival rate of patients with PDAC is extremely low and has not improved in recent decades, accurate diagnosis at an early stage significantly improves the prognosis. Recently, Hanaeda et al. reported an improved 5-year-survival of up to 80% when the cancer size was < 10 mm [4, 43]. Finally, because the test is based on the qPCR assay, it can be easily and quickly performed at any laboratory; improving the convenience of the diagnostic process both for physicians and patients.

This study has several limitations. The regression equation markers were developed from the development cohort, their efficacy was determined in a separate verification cohort, and the exact functional role of the markers was not well investigated. Although we found that IL-7R, PLD4, and ID3 are differently expressed in human PBMC of PDAC patients, the functional role of marker-expressing immune cells, spatiotemporal relationship of the markers in tumor development, and precise mechanisms for marker upregulation remain unclear. As interest in the immune environment of tumor progression is still in its infancy for most malignancies, these unsolved issues should be addressed in the future. In addition, whether tumor-induced immunity suppresses tumorigenesis or supports tumor growth is context-dependent; ultimately, the global immune landscape beyond the tumor is significantly altered during tumor progression [44]. Over the last few decades, immunotherapy has revolutionized cancer therapy, such as anti-CTLA4, anti-PD1, and anti-PDL1. Therefore, although our PBMC marker data are helpful and may be useful for clinical PDAC diagnosis, the levels of the markers should be monitored and determined in a time-dependent manner. This will allow the changes in marker levels to be fully understood and the true value of the regression equation of the three-marker combination to be determined.

## Conclusion

We found PDAC-specific PBMC markers that are upregulated in PDAC cases and can be easily measured by small-volume blood sampling. The logistic equation developed by combining PBMC-related immune markers from patients with PDAC aided the differential diagnosis of indeterminate PDAC cases, reduced FNR of abdominal CT and CA19-9 level, and improved predictability for PDAC diagnosis.

## Supplementary Information

Below is the link to the electronic supplementary material.

Supplementary file 1 (DOCX 70 KB)

## Data Availability

The data presented in this study are available on request from the corresponding author.

## Abbreviations

AUC: Area under the curve
CI: Confidence interval
CT: Computed tomography
DEG: Differentially expressed gene
EUS: Endoscopic ultrasound
FNR: False-negative rate value
GI: Gastrointestinal
ID3: Inhibitor of DNA binding 3
IL-7R: Interleukin-7 receptor
mRNA-seq: MRNA sequencing
NPV: Negative predictive value
PBMC: Peripheral blood mononuclear cells
PDAC: Pancreatic ductal adenocarcinoma
PLD4: Phospholipase D4
PPV: Positive predictive value
qPCR: Quantitative PCR
RMA: Robust multi-average
RNA-seq: RNA-sequencing
scRNA-seq: Single-cell RNA-seq
WT: Whole transcript
