# Supplementary material for: Improved predictability of pancreatic ductal adenocarcinoma diagnosis using a blood immune cell biomarker panel developed from bulk mRNA sequencing and single-cell RNA-sequencing
Source: Cancer Immunol Immunother. 2023 May 10;72(8):2757–68. doi: 10.1007/s00262-023-03458-8 (PMC10361912; doi:10.1007/s00262-023-03458-8)
Supplement: Supplementary file 1 — (DOCX 70 KB) [file 262_2023_3458_MOESM1_ESM.docx]

**Supplementary Appendix**

This appendix has been provided by the authors to give readers additional information regarding their work.

Supplement to: Improved predictability of pancreatic ductal adenocarcinoma diagnosis using a blood immune cell biomarker panel developed from bulk mRNA sequencing and single-cell RNA-sequencing

Sung Ill Jang, Hyung Keun Lee, Eun-Ju Chang, Somi Kim, So Young Kim, In Young Hong, Jong Kyoung Kim, Hye Sun Lee, Juyeon Yang, Jae Hee Cho, Dong Ki Lee

**Contents**

**1. Supplementary Results: page**

**Supplementary Table 1: page 2**

**Supplementary Table 2: page 3**

**Supplementary Figure 1: page 4**

**Supplementary Table 3: page 5**

**Supplementary Table 4: page 6**

**Supplementary Table 5: page 7**

**Supplementary Table 6: page 8**

**Supplementary Table 1. Primers used in the qRT-PCR assays**

| **Gene** | **Primer** | **Sequence** |
| --- | --- | --- |
| *hIL-7RA* | Forward | 5’-CTG GAG AAA GTG GCT ATG CTC-3’ |
|  | Reverse | 5’-ACA TCT GGG TCC TCA AAA GC-3’ |
|  | Probe-FAM | 5’-CAG TTG GAA GTG AAT GGA TCG CAG C-3’ |
| *hPLD4* | Forward | 5’-CAA ATT CTG GGT TGT GGA TGG-3’ |
|  | Reverse | 5’-AGG TGG CTG CAG TTA TAG ATG-3’ |
|  | Probe-FAM | 5’-TCT GAC GCA GGT GAA GGA GCT TG-3’ |
| *hID3* | Forward | 5’-GAC TAC ATT CTC GAC CTG CAG-3’ |
|  | Reverse | 5’-TCG TTG GAG ATG ACA AGT TCC-3’ |
|  | Probe-FAM | 5’-CTC GGC TGT CTG GAT GGG AAG G-3’ |
| *hGAPDH* | Forward | 5’-CCA AGG TCA TCC ATG ACA ACT-3’ |
|  | Reverse | 5’-ATC ACG CCA CAG TTT CCC-3’ |
|  | Probe-Joe | 5’ATC ACT GCC ACC CAG AAG ACT GTG-3’ |

**Supplementary Table 2. Primary antibodies and methods used for Immunohistochemistry**

| **Antibody** | **Clone** | **Manufacturer** | **Method** | **Detection** |
| --- | --- | --- | --- | --- |
| IL-7RA | Rabbit Polyclonal | LS Bio  (LS-B2830) | BenchMark Ultra IHC/ISH System  (Ventana Medical Systems) | 1. OptiView DAB IHC  Detection Kit  (Ventana Medical Systems)  2. ultraView Universal Alkaline Phosphatase Red Detection Kit  (Ventana Medical Systems) |
| PLD4 | Rabbit Polyclonal | Sigma-Aldrich  (HPA051512) |  |  |
| ID3 | Rabbit Polyclonal | Sigma-Aldrich  (HPA024677) |  |  |
| CD20 | Mouse monoclonal  (L26) | Leica Biosystems |  |  |
| CD3 | Rabbit monoclonal  (2GV6) | Ventana Medical Systems |  |  |

^a^ IL-7RA+CD3 double staining

^b^ PLD4+CD20 double staining

^c^ ID3+CD3 double staining

**Supplementary Figure 1. Bar plot showing inferred cell type composition in RNA-seq per sample.**


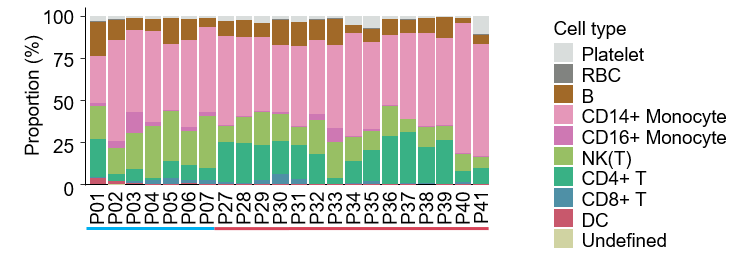


**Supplementary Table 3.** **Characteristics of the study population in development cohort.**

|  | **Pancreatic cancer**  **(n=50)** | **Control**  **(n=61)** | **High risk group**  **(n=56)** | **Benign pancreatic disease**  **(n=51)** | **Other GI malignancy**  **(n=54)** |
| --- | --- | --- | --- | --- | --- |
| **Age**, years (mean ± SD) | 68.5 ± 10.9 | 52.0 ± 12.5**^†^** | 63.9 ± 10.6**^†^** | 54.9 ± 16.2^†^ | 66.9 ± 12.3 |
| **Male : female** | 26:24 | 29:32 | 26:30 | 34:17 | 30:24 |
| **BMI**, kg/m^2^ (mean ± SD) | 22.5 ± 2.7 | 24.5 ± 4.0^†^ | 23.5 ± 2.5 | 25.5 ± 4.3^†^ | 23.9 ± 3.3^†^ |
| **DM**, n (%) | 24 (48.0) | NA | 13 (23.2) | 12 (23.6) | 14 (25.9) |
| **WBC**, count/uL (mean ± SD) | 7496.4 ± 3568.9 | 6228.7 ± 1952.1^†^ | 6159.5 ± 1808.2^†^ | 7295.9 ± 3479.2 | 6868.9 ± 2747.7 |
| **Neutrophil** | 5107.0 ± 3181.2 | 3404.4 ± 1342.6^†^ | 3633.4 ± 2991.3^†^ | 4721.8 ± 3427.4 | 5781.8 ± 9791.2 |
| **Lymphocyte** | 1592.5 ± 699.9 | 2170.2 ± 635.0^†^ | 2036.1 ± 763.3^†^ | 1836.9 ± 706.9 | 1531.5 ± 571.3 |
| **Monocyte** | 583.9 ± 304.8 | 438.2 ± 171.1^†^ | 465.2 ± 160.2^†^ | 551.4 ± 228.9 | 601.5 ± 287.1 |
| **Eosinophil** | 142.6 ± 121.8 | 169.2 ± 158.8 | 190.7 ± 164.2 | 175.6 ± 198.9 | 213.3 ± 192.8^†^ |
| **Basophil** | 35.4 ± 18.5 | 41.1 ± 22.4 | 40.5 ± 23.3 | 36.1 ± 19.1 | 36.3 ± 18.5 |
| **Platelet** count 10^3^/ μL (mean ± SD) | 235.8 ± 88.9 | NA | 238.5 ± 90.6 | 228.1 ± 73.5 | 226.9 ± 87.6 |
| **Protein**, g/dL (mean ± SD) | 6.7 ± 1.1 | 7.1 ± 0.5^†^ | 6.9 ± 0.5 | 6.9 ± 0.9 | 6.4 ± 0.8 |
| **Albumin**, g/dL (mean ± SD) | 3.9 ± 0.5 | 4.3 ± 0.6^†^ | 4.2 ± 0.4^†^ | 4.1 ± 0.6 | 3.6 ± 0.7^†^ |
| **Bilirubin**, mg/dL (mean ± SD) | 2.5 ± 4.0 | 0.7 ± 0.3^†^ | 0.7 ± 0.3^†^ | 1.1 ± 0.7^†^ | 3.4 ± 6.1 |
| **AST**, IU/L (mean ± SD) | 94.9 ± 155.2 | 26.2 ± 12.0^†^ | 27.5 ± 10.5^†^ | 45.0 ± 40.3^†^ | 48.7 ± 54.3^†^ |
| **ALT**, IU/L (mean ± SD) | 112.9 ± 176.5 | 22.2 ± 13.7^†^ | 22.9 ± 14.1^†^ | 52.2 ± 64.6^†^ | 57.9 ± 85.3^†^ |
| **CRP**, mg/L (mean ± SD) | 20.5 ± 43.4 | NA | 3.5 ± 15.7^†^ | 38.1 ± 63.6 | 19.8 ± 38.1 |
| **CEA,** ng/L (mean ± SD) | 70.1 ± 402.5 | 1.8 ± 0.9 | 3.1 ± 3.0 | 2.5 ± 2.0 | 173.8 ± 840.2 |
| **CA19-9**, U/mL (mean ± SD) | 2254.6 ± 5357.2 | 7.2 ±6.0^†^ | 23.7 ± 51.5^†^ | 11.0 ± 15.2^†^ | 1059.3 ± 3101.2^†^ |
| **IL-7R**, (mean ± SD) | 1235.8 ± 597.2 | 947.7 ± 396.4^†^ | 1188.6 ± 566.7 | 938.6 ± 578.4^†^ | 929.6 ± 633.9^†^ |
| **PLD4,** (mean ± SD) | 11.7 ± 6.3 | 26.4 ± 7.8^†^ | 18.7 ± 8.6^†^ | 14.9 ± 8.9 | 15.1 ± 9.7^†^ |
| **ID3,** (mean ± SD) | 10.4 ± 6.8 | 7.0 ± 3.8^†^ | 7.6 ± 4.5^†^ | 7.9 ± 7.8^†^ | 5.8 ± 4.7^†^ |

^GI, gastrointestinal; SD, standard deviation; BMI, body mass index; DM, diabetes mellitus; NA, non-available; WBC, whole blood cell; AST, aspartate transaminase; ALT, alanine transaminase; CRP, c-reactive protein; CEA, carcinoembryonic antigen; CA19-9, carbohydrate antigen 19-9; IL-7R, Interleukin-7 receptor, PLD4, Phospholipase D4; ID3, inhibitor of DNA binding 3^

**^†, P-value<0.005 comparing with pancreatic cancer^**

**Supplementary Table 4.** **CA 19-9, IL-7R, PLD4 and CA19-9 between pancreatic cancer and non-pancreatic cancer.**

| Variable (mean ± SD) | Pancreatic cancer (n=50) | Non-pancreatic cancer (n=222) | *p-*value | Univariable model | |  | Multivariable model | |
| --- | --- | --- | --- | --- | --- | --- | --- | --- |
|  |  |  |  | OR (95% CI) | *p-*value |  | OR(95% CI) | *p-*value |
| CA19-9* | 2254.6 ± 5357.3 | 226.3 ± 1446.4 | 0.0106 | 1.000 (1.000-1.000) | 0.0096 |  |  |  |
| IL7R† | 1235.8 ± 597.2 | 1013.7 ± 580.6 | 0.0157 | 0.908 (0.872-0.946) | <.0001 |  | 0.795(0.736-0.859) | <.0001 |
| PLD4† | 11.7 ± 6.3 | 19.1 ± 9.9 | <.0001 | 1.001 (1.000-1.001) | 0.0179 |  | 1.001(1.000-1.002) | 0.0025 |
| ID3† | 10.4 ± 6.8 | 7.3 ± 5.8 | 0.001 | 1.075 (1.026-1.126) | 0.0022 |  | 1.144(1.059-1.235) | 0.0006 |

^CA19-9, carbohydrate antigen 19-9; IL-7R, Interleukin-7 receptor, PLD4, Phospholipase D4; ID3, inhibitor of DNA binding 3; SD, standard deviation^

***, ^U/ml in serum^**

**†^, mRNA level in PBMC^**

**Supplementary Table 5.** **Characteristics of the study population in validation cohort.**

|  | **PDAC-positive group**  **(n=43)** | **PDAC-suspicious group (n=113)** | **P-value** |
| --- | --- | --- | --- |
| **Age**, years (mean ± SD) | 64.9 ± 13.6 | 61.3 ± 15.7 | 0.175 |
| **Male : female** | 25:18 | 61:52 | 0.643 |
| **BMI**, kg/m^2^ (mean ± SD) | 23.3 ± 3.3 | 23.4 ± 3.5 | 0.773 |
| **DM**, n (%) | 16 (37.2) | 33 (29.2) | 0.293 |
| **WBC**, count/uL (mean ± SD) | 7361.6 ± 2818.9 | 6911.5 ± 3061.1 | 0.404 |
| **Neutrophil** | 6613.5 ± 11886.3 | 4751.5 ± 2913.8 | 0.274 |
| **Lymphocyte** | 1531.2 ± 816.2 | 1501.1 ± 575.4 | 0.797 |
| **Monocyte** | 573.3 ± 247.7 | 548.5 ± 593.8 | 0.792 |
| **Eosinophil** | 167.2 ± 172.9 | 179.8 ± 157.6 | 0.664 |
| **Basophil** | 34.2 ± 19.7 | 32.2 ± 18.4 | 0.557 |
| **Platelet** count 10^3^/ μL (mean ± SD) | 233.1 ± 88.1 | 245.3 ± 109.5 | 0.511 |
| **Protein**, g/dL (mean ± SD) | 6.7 ± 0.7 | 6.6 ± 0.6 | 0.354 |
| **Albumin**, g/dL (mean ± SD) | 3.8 ± 0.6 | 3.9 ± 0.4 | 0.589 |
| **Bilirubin**, mg/dL (mean ± SD) | 3.3 ± 5.1 | 1.3 ± 1.9 | 0.016 |
| **AST**, IU/L (mean ± SD) | 76.6 ± 108.0 | 46.1 ± 70.9 | 0.091 |
| **ALT**, IU/L (mean ± SD) | 73.6 ± 119.4 | 51.1 ± 89.6 | 0.267 |
| **CRP**, mg/L (mean ± SD) | 24.0 ± 40.2 | 28.4 ± 60.5 | 0.609 |
| **CEA,** ng/L (mean ± SD) | 22.8 ± 47.4 | 9.5 ± 58.6 | 0.148 |
| **CA19-9**, U/mL (mean ± SD) | 2671.9 ± 5431.2 | 908.2 ± 3760.7 | 0.055 |
| **IL-7R**, (mean ± SD) | 1311.7 ± 823.5 | 1123.2 ± 653.3 | 0.137 |
| **PLD4,** (mean ± SD) | 13.4 ± 7.9 | 15.2 ± 8.3 | 0.229 |
| **ID3,** (mean ± SD) | 10.5 ± 7.2 | 10.3 ± 6.6 | 0.848 |
| **Biomarkers panel,** (mean ± SD) | 0.3347 ± 0.1756 | 0.2318 ± 0.1669 | 0.001 |
| **Final diagnosis, PDAC, n (%)** | 43 (100) | 33 (29.2) | < 0.001 |

PDAC, pancreatic ductal adenocarcinoma; SD, standard deviation; BMI, body mass index; DM, diabetes mellitus; NA, non-available; WBC, whole blood cell; AST, aspartate transaminase; ALT, alanine transaminase; CRP, c-reactive protein; CEA, carcinoembryonic antigen; CA19-9, carbohydrate antigen 19-9; IL-7R, Interleukin-7 receptor, PLD4, Phospholipase D4; ID3, inhibitor of DNA binding 3

**Supplementary Table 6.** Abnormal findings on abdominal computed tomography in suspicious PDAC patients

| Computed tomography findings | No. of patients (%) |
| --- | --- |
| Focal alteration of parenchymal attenuation | 76 (67.3) |
| Pancreatic duct dilatation | 39 (34.5) |
| Cystic lesion with high malignant stigma | 28 (24.8) |
| Parenchymal atrophy | 27 (23.9) |
| Contour abnormality of pancreatic parenchyma | 18 (15.9) |
| Peripancreatic lymphadenopathy | 18 (15.9) |
| Bile duct dilatation | 12 (10.6) |
| Double-duct sign | 12 (10.6) |
| Pancreatic duct interruption | 1 (0.9) |
